# Genomic analysis reveals complex population structure within the smooth newt, *Lissotriton vulgaris*, in Central Europe

**DOI:** 10.1002/ece3.10478

**Published:** 2023-08-31

**Authors:** Dávid Herczeg, Gemma Palomar, Piotr Zieliński, Isolde van Riemsdijk, Wiesław Babik, Róbert Dankovics, Bálint Halpern, Milena Cvijanović, Judit Vörös

**Affiliations:** ^1^ ELKH‐ELTE‐MTM Integrative Ecology Research Group Budapest Hungary; ^2^ Department of Systematic Zoology and Ecology, Institute of Biology ELTE Eötvös Loránd University Budapest Hungary; ^3^ Department of Genetics, Physiology, and Microbiology, Faculty of Biological Sciences Complutense University of Madrid Madrid Spain; ^4^ Institute of Environmental Sciences Faculty of Biology, Jagiellonian University Kraków Poland; ^5^ Department of Biology Lund University Lund Sweden; ^6^ Savaria Museum Szombathely Hungary; ^7^ MME Birdlife Hungary Budapest Hungary; ^8^ Institute for Biological Research “Siniša Stanković”, National Institute of the Republic of Serbia University of Belgrade Belgrade Serbia; ^9^ Department of Zoology Hungarian Natural History Museum Budapest Hungary

**Keywords:** barrier effect, Carpathian Basin, evolutionary lineages, genetic diversity, phylogeography, Salamandridae

## Abstract

Species with wide‐range distributions usually display high genetic variation. This variation can be partly explained by historical lineages that were temporally isolated from each other and are back into secondary reproductive contact, and partly by local adaptations. The smooth newt (*Lissotriton vulgaris*) is one of the most widely distributed amphibians species across Eurasia and forms a species complex with a partially overlapping distribution and morphology. In the present study, we explored the population genomic structure of smooth newt lineages in the Carpathian Basin (CB) relying on single‐nucleotide polymorphisms. Our dataset included new and previously published data to study the secondary contact zone between lineages in the CB and also tested for the barrier effect of rivers to gene flow between these lineages. We confirmed the presence of the South *L. v. vulgaris* Lineage distributed in Transdanubia and we provided new distribution records of *L. v. ampelensis* inhabiting the eastern territories of the CB. High genetic diversity of smooth newts was observed, especially in the North Hungarian Mountains and at the interfluves of the main rivers in the South with four distinct lineages of *L. v. vulgaris* and one lineage of *L. v. ampelensis* showing a low level of admixture with the spatially closest *L. v. vulgaris* lineage. Moreover, admixture detected at the interfluve of the main rivers (i.e. Danube and Tisza) suggested a secondary contact zone in the area. Finally, we found that the river Danube has a very weak effect on population divergence, while the river Tisza is a geographical barrier limiting gene flow between smooth newt lineages. As the range boundaries of *L. v. vulgaris* and *L. v. ampelensis* in the CB coincide with the river Tisza, our study underpins the influence of rivers in lineage diversification.

## Graphical abstract

In the present study, we explored the population genomic structure of smooth newts in the Carpathian Basin (CB) relying on single‐nucleotide polymorphism. High genetic diversity of smooth newts was observed with four distinct lineages of *L. v. vulgaris* and one consistent lineage of *L. v. ampelensis* showing a low level of admixture with the spatially closest *L. v. vulgaris* lineage. As the range boundaries of *L. v. vulgaris* and *L. v. ampelensis* in the CB coincide with the river Tisza, our study underpins the influence of rivers in lineage diversification.

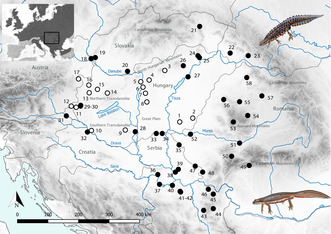

## 1 INTRODUCTION

Generally, species distributions are relatively close to equilibrium with the present climate; however, animals with varying dispersal abilities suggests some degree of discrepancy from that equilibrium (Araújo & Pearson, 2005). In Europe, the current distributions of the herpetofauna are determined more by their proximity to the three major glacial refugial areas, that is, the Iberian, Appenin and Balkan peninsulas (Hewitt, 2004; Taberlet et al., 1998), than by present climate gradients (Araújo & Pearson, 2005). Europe's herpetofauna is considered well‐explored (Speybroeck et al., 2020). Nevertheless, with the advance of molecular research, coupled with the application of genomic technologies, substantial cryptic diversity has been unveiled—particularly evident in the three above mentioned regions (Velo‐Antón et al., 2023). Multiple lines of fossil evidence suggested that the three major refugial areas must have been complemented with several extra‐Mediterranean refugia, for example, in Central Europe including the Carpathian Basin (CB) and the Dordogne in south‐western France (Sommer & Benecke, 2005; Sommer & Nadachowski, 2006). Furthermore, increasing evidence confirms that the European southern and eastern refugia were supplemented by multiple cryptic refugia in northern Europe during the Late Pleistocene (Rowe et al., 2006; Stewart & Lister, 2001; Tzedakis et al., 2013). The discovery of these cryptic refugia entailed growing interest and extensive research of Central European fauna leading to the detection of unexpected cryptic diversity, particularly along the southern borders with the Balkan Peninsula (Varga, 2019).

The CB is a complex transitional zoogeographic and climatic area between the Carpathian Mountains and the western Alpine domain. Due to its unique geographical characteristics, microclimate and hydrological conditions, the CB provided extra‐Mediterranean glacial refugia for many temperate species during the cold periods of the last glaciation (Schmitt, 2007; Schmitt & Varga, 2012; Sommer & Nadachowski, 2006; Varga, 2010; Vörös et al., 2021). In the case of amphibians, cryptic diversity and the evidence of Central European refugia were supported by inferences based on genetic data from *Bombina* toads (Fijarczyk et al., 2011; Hofman et al., 2007; Spolsky et al., 2006; Vörös et al., 2006), the moor frog, *Rana arvalis* (Babik et al., 2004), the *Triturus* newts (Vörös et al., 2016; Wielstra et al., 2013), the Carpathian newt, *Lissotriton montandoni* (Wielstra et al., 2017), and alpine newts (Robbemont et al., 2023).

The smooth newt, *L. vulgaris*, sensu lato has a parapatric distribution with a large range across Eurasia (Arntzen, Kuzmin, Beebee, et al., 2009). At the intraspecific level, *L. vulgaris* is differentiated into at least seven subspecies and forms a speciation continuum of evolutionary lineages with partially overlapping geographical ranges and morphology (Raxworthy, 1990). The northern part of this distribution is inhabited by the wide‐ranging *L. v. vulgaris* subspecies which was recently separated, using multilocus data, into North *L. v. vulgaris* Lineage and South *L. v. vulgaris* Lineage (Pabijan et al., 2017). The morphologically well‐characterized *L. v. graecus* inhabits the southern Balkan Peninsula, *L. v. kossingwi* is restricted to a small area along the Black Sea coast east of Istanbul, *L. v. schmidtlerorum* dominating western Anatolia and the coast of the Marmara Sea, while *L. v. lantzi* is limited to the northern Caucasus and parts of Transcaucasia. Recently, the aforementioned four southern subspecies were elevated up to the species level, that is, *L. graecus*, *L. kosswigi*, *L. lantzi* and *L. schmidtleri* according to Pabijan et al. (2017). Furthermore, the Apennine Peninsula and adjacent territories are inhabited by *L. v. meridionalis*, and ultimately *L. v. ampelensis* has a broader distribution in Transylvania reaching the Carpathians and the Harghita Upland on the east but its presence has never been confirmed in the eastern part of the CB. For the visual presentation of the distribution of the morphological species and subspecies see Figure 1 in Pabijan et al. (2017). The parapatric distribution of Central European lineages supports a relatively high level of gene flow among them. The genetic cluster of *L. v. ampelensis* contains two distinct morphological forms and extensive genetic exchange with the North *L. v. vulgaris* Lineage and *L. montandoni* was confirmed (Pabijan et al., 2017; Zieliński et al., 2016, 2019). Gene flow between the South *L. v. vulgaris* Lineage and the neighbouring geographical lineage of *L. v. ampelensis*, and between the North *L. v. vulgaris* Lineage and *L. v. meridionalis* was also detected (Pabijan et al., 2017). In this study, we focus on the fine‐scale distribution and population structure of two evolutionary lineages, the South *L. v. vulgaris* Lineage and the *L. v. ampelensis* of smooth newts in the CB, where we suspect a secondary contact zone between them (Pabijan et al., 2017).

**FIGURE 1:**
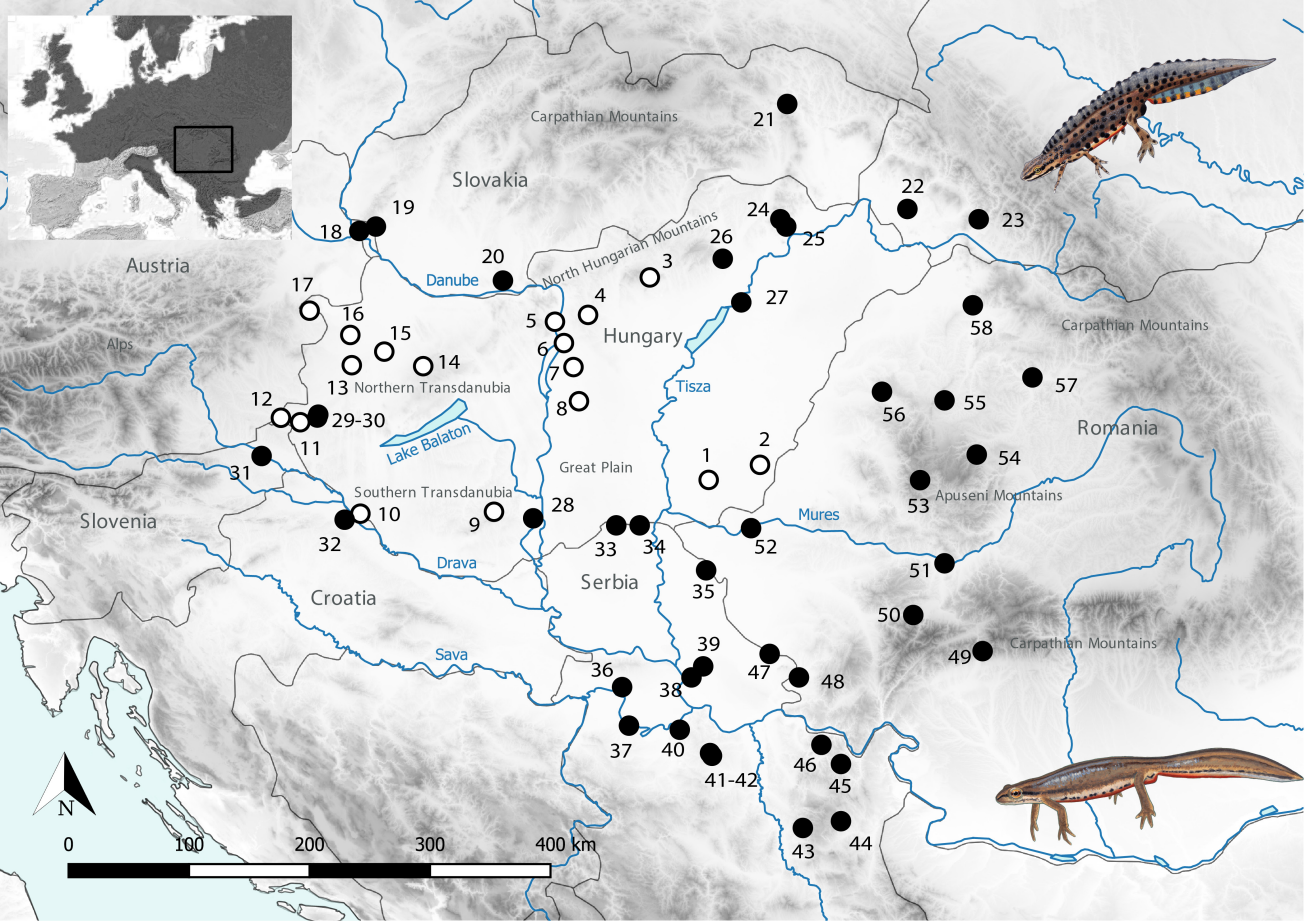
Sampling of *Lissotriton vulgaris vulgaris* and *L. v. ampelensis* in the Carpathian Basin. White dots represent localities sampled for this study, while black dots represent localities added from Wielstra et al. (2018). Serbian samples originated from the Institute for Biological Research ‘Siniša Stanković’ University of Belgrade (Džukić et al., 2015). Locality IDs correspond with Population IDs in Table S1. Sample size is indicated in Table S1. The top left insert shows the location of the study area within Europe (black frame) and the distribution of *L. vulgaris* in Europe (dark grey shading; modified from AmphibiaWeb, 2021). Drawings of *L. v. vulgaris* male and female, courtesy of Márton Zsoldos.

Landscape features are crucial determinants of population structuring because they may shape the dispersal of species, influencing gene flow leading to a divergence between their populations, and ultimately driving speciation events (Manel et al., 2003). Rivers can act as boundaries for terrestrial (Figueiredo‐Vázquez et al., 2021; Moraes et al., 2016; Vörös et al., 2006; Wang et al., 2015) and semiaquatic amphibians (Figueiredo‐Vázquez et al., 2021), and as dispersal corridors for fully aquatic species (Burbrink et al., 1998; Vörös et al., 2016).

The Balkan Peninsula was proposed as the primary source of genetic diversity of the *L. vulgaris* complex and served as a refugium for consequent recolonization of central and northern Europe during the last glacial period (Babik et al., 2005; Pabijan et al., 2015). Nonetheless, the existence of multiple, spatially distinct refugia was suggested north and east of the Balkans, which may have conserved some fraction of the Pleistocene genetic diversity of smooth newts (Babik et al., 2005). For instance, the distribution of mitochondrial DNA (mtDNA) clades in *L. vulgaris* and *L. montandoni*, and gene flow between the South *L. v. vulgaris* Lineage and *L. kosswigi* suggests that the CB and Turkey were probably among these refugia (Babik et al., 2005; Nadachowska & Babik, 2009). It was presumed that the secondary contact between the evolutionary lineages of smooth newts is situated in the eastern part of the CB (Pabijan et al., 2017). Therefore, we use population genomics to (i) investigate the current distribution and population structure of *L. vulgaris* lineages in the CB, (ii) explore the putative contact zone between the South *L. v. vulgaris* Lineage and *L. v. ampelensis* in the eastern part of CB and (iii) determine the effect of the geographical boundaries (i.e. the river Danube and the river Tisza) potentially shaping the population structure of smooth newts in the study area.

## 2 MATERIALS AND METHODS

### 2.1 Sample collection, DNA extraction and SNP library preparation

In total, 98 *L. vulgaris* tail clips from 17 localities (each locality represents one distinct population) were collected in Hungary during the spring of 2020 (Table S1, Figure 1). Additionally, 41 DNA sequences from 41 localities (each locality represents a distinct population) previously published by Wielstra et al. (2018) complemented our dataset to get a fine‐scale coverage of the contact zone between the South *L. v. vulgaris* Lineage and *L. v. ampelensis* in the CB (Table S1, Figure 1). In the following, we use the term population to refer both individuals (in case of additional samples) and localities (in case of fresh sampling) because in this case, they are identical. DNA from tail clip tissues was extracted with the Wizard Genomic DNA Purification Kit according to the manufacturer's instructions (Promega). Fragments of 1233 genes were amplified using Molecular Inversion Probes (MIPs) following the protocol from Niedzicka et al. (2016). MIPs are single‐stranded DNA molecules containing on their ends sequences complementary to two regions flanking the target (i.e. the arms), which is around 100 bp. The description of the MIP is detailed in Zieliński et al. (2019). MIPs were pooled equimolarly and hybridized to the genomic DNA. Following the gap‐filling and the ligation, resulting circular DNA molecules were used as template for the PCR. After sequencing in an Illumina MiSeq platform and considering that the same laboratory protocol was used to develop both datasets (i.e. our dataset and data of Wielstra et al., 2018), both sequencing data were joint. Then, all fragments were mapped to references (Zieliński et al., 2019) and the arms were trimmed. Single‐nucleotide polymorphism (SNP) calling was performed with UnifiedGenotyper of GATK 3.6 (DePristo et al., 2011; McKenna et al., 2010). Individual genotypes with sequencing depth ≤8 and genotype quality below 20 were treated as missing. Only biallelic SNPs with <50% of missing data and minor allele frequency ≥2% were used in the downstream analyses. Furthermore, three samples (LvP72, LvP06 and LvP98) were discarded from the analysis due to an excess of missing data, thus further analyses were conducted with 136 individuals from 58 populations in total.

### 2.2 Population structure

Principal component analysis (PCA) was performed in Plink 1.9 (Chang et al., 2015) to investigate the structure of the genetic variation of the data. In addition, ADMIXTURE software (Alexander et al., 2009) was applied to estimate the genetic ancestry of each sample. ADMIXTURE's cross‐validation procedure (Alexander & Lange, 2011) was performed to distinguish between the number of ancestral populations in the dataset. This method also provided the estimates of pairwise *F*_st_ between ancestral populations. The mean value of the genetic ancestry per population was used to construct pie charts. All maps were created in QGIS 3.16 (QGIS, 2021). Expected heterozygosity (HE) was computed in Arlequin 3.5 (Excoffier & Lischer, 2010). We visualized the geographical distribution of HE using heatmap render implemented in QGIS. We performed this analysis both with the whole dataset and with a single SNP per random locus and the outcome was similar (99% correlation between the two analyses), therefore we kept a heatmap showing the distribution of HE for the whole dataset. The layer with water bodies was downloaded from the European Environment Agency (www.eea.europa.eu/data‐and‐maps/data/wise‐large‐rivers‐and‐large‐lakes). Finally, an unrooted phylogenetic tree was reconstructed based on the genetic distance matrix (*F*_st_) and neighbour‐joining method implemented in MEGA software (Tamura et al., 2021) to visualize the relationships among lineages.

### 2.3 Testing the effect of rivers as barriers to gene flow

To explore whether the data showed signs of a barrier to gene flow posed by the Danube and Tisza rivers, we first visualized the relationship between *F*_st_ and geographical distance in relation to the side of the Danube and the Tisza, by using pairwise geographical and *F*_st_ distances. We then coloured the pairwise comparisons between populations on the same or different sides of the Danube and Tisza (Figure 6), following Bradburd et al. (2013).

To further understand the effect of the rivers on genetic distance between *L. vulgaris* populations, we performed a Bayesian Estimation of Differentiation in Alleles by Spatial Structure and Local Ecology (i.e. BEDASSLE) analysis with the ‘BEDASSLE’ package in R (Bradburd et al., 2013). BEDASSLE can be used to fit effect sizes for each environmental predictor (i.e. the Danube and Tisza rivers) and geographical distance, based on the matrices of dissimilarity for geography and ecology, genetic data (in our case a random selection of one SNP per MIP fragment to ensure independence between SNPs) and sample sizes. The BEDASSLE model assumes that populations are at migration–drift equilibrium such that the allele presence/absence can be modelled based on the geographical distance between populations (Bradburd et al., 2013). Geographical distance was normalized. We ran BEDASSLE with the beta‐binomial mode, which prevents outlier populations from having an inappropriate influence on the effect size estimates of the predictor variables, adapting a script available from GitHub (Grieneisen et al., 2019). The final model was run with two million generations, samples recorded every 1000 generations, and a 200 sample burn‐in was removed (based on initial trace plots). Trace plots without burn‐in (Figure S2A) were assessed for appropriate mixing of the model. We ran 1000 posterior predictive samples to determine that the model was a good fit for the data (Figure S2B).

## 3 RESULTS

After filtering, a total of 4124 SNPs from 1065 genes were used for the analysis. The mean percentage of missing data was 6.6% and the average coverage depth was around 63 reads per sample per locality.

### 3.1 Population structure

**FIGURE 2:**
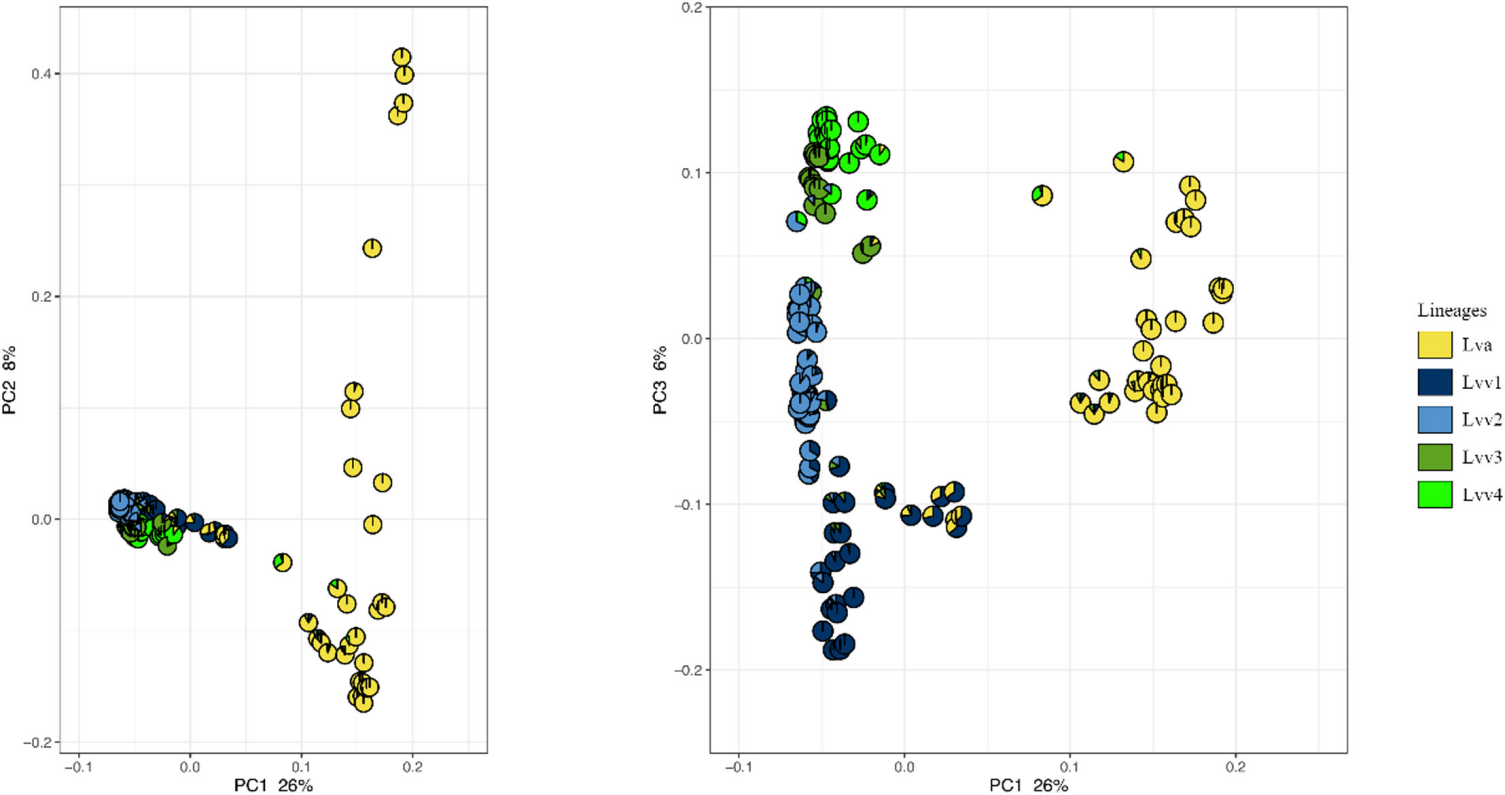
Scatterplot based on PCA with pie charts showing the admixture in each individual. The left plot is PC1 versus PC2, while the right plot is PC1 versus PC3. Lva, *Lissotriton vulgaris ampelensis*; Lvv1–Lvv4, *L. v. vulgaris* cluster 1–4.

**FIGURE 3:**
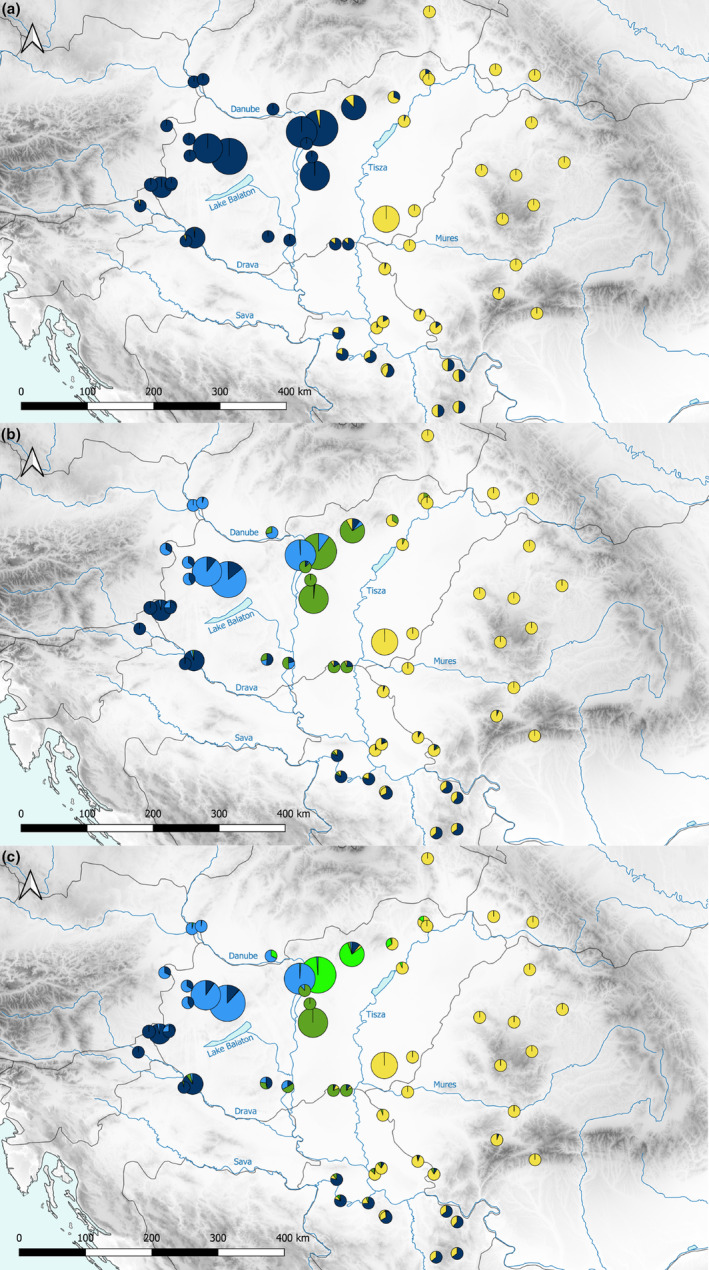
ADMIXTURE's cross‐validation results for *K* = 2 (a), *K* = 4 (b) and *K* = 5 (c). Each colour represents one cluster. The size of the pie charts are corresponding to the sample size of each population. Lva, *Lissotriton vulgaris ampelensis*; Lvv1–Lvv4, *L. v. vulgaris* cluster 1–4. Lineages and colours correspond with Figure 2.

The distribution of the samples along the principal component (PC) 1 (explaining 26% of the variance), 2 (8%) and 3 (6%) is presented in Figure 2. ADMIXTURE's cross‐validation procedure supported four ancestral clusters (CV error = 0.352); however, the difference from two to five ancestral clusters was minimal (0.352–0.368). The two clusters found corresponded to the South *L. v. vulgaris* Linage and *L. v. ampelensis*. With increasing the number of ancestral clusters, *L. v. ampelensis* remained stable while the South *L. v. vulgaris* Lineage were divided into two to four (Lvv1–4) additional clusters (Figure 3a–c). This was also supported by the distribution of samples within PC1–PC3 (Figure 2). While PC1 showed the difference between lineages, PC3 demonstrated the differences among the South *L. v. vulgaris* clusters (Figure 2). An admixture between the South *L. v. vulgaris* Lineage and *L. v. ampelensis* and also clusters found among the South *L. v. vulgaris* Lineage was detected (Figures 2 and 3b,c).

**FIGURE 4:**
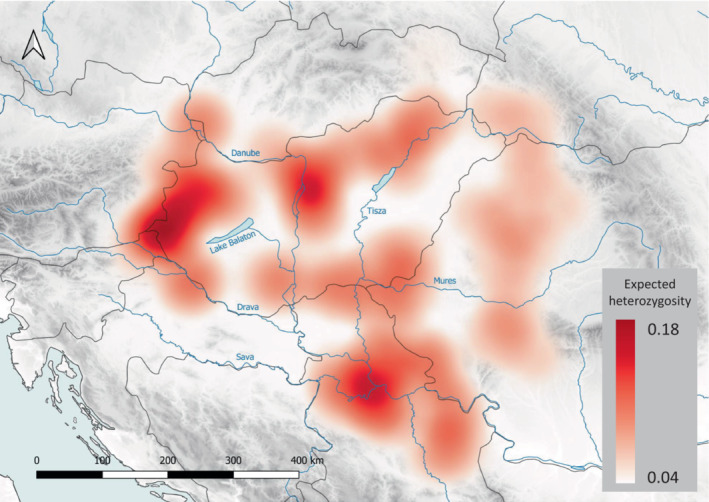
Heat map of expected heterozygosity visualizing genetic diversity among *Lissotriton vulgaris* populations in the Carpathian Basin.

**FIGURE 5:**
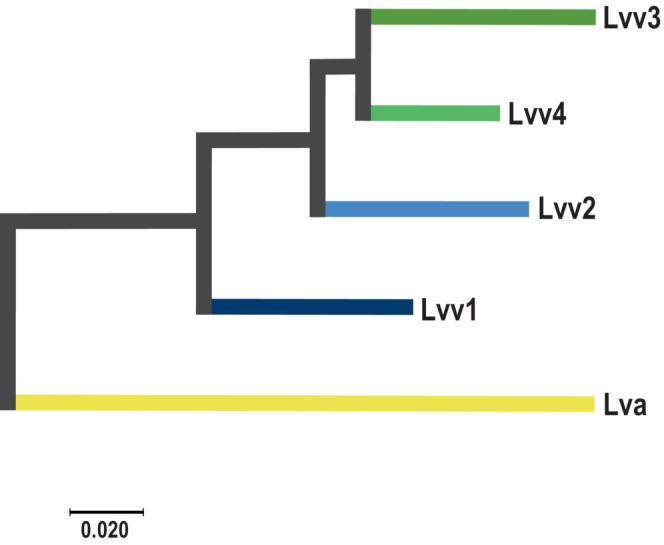
Unrooted genetic distance tree featuring relationships among *Lissotriton vulgaris ampelensis* (Lva) and *L. v. vulgaris* (Lvv1–Lvv4) clusters.

The *F*_st_ statistics showed high differentiation (0.266) between *L. v. ampelensis* and the South *L. v. vulgaris* Lineage, and also between *L. v. ampelensis* and all the four South *L. v. vulgaris* Lineage (Lvv1–4) clusters (0.266–0.31). Lower differentiation was found among the South *L. v. vulgaris* Lineage clusters (0.103–0.157), cluster Lvv1 being the most differentiated from the others (0.133–0.157; see Table 1). The HE ranged from 0.04 (Locality 21, Lva) to 0.17 (Locality 35, Lva) with a mean of 0.13 (SD = 0.02) and median of 0.14 (Figure 4, Figure S1). Lowest HE in Locality 21 was most probably due to the high number of missing genotypes (52%) in this sample. The differences between populations in HE were low (Figure S1). However, there was a clear geographical pattern of its distribution as evidenced by the heat map (Figure 4). The genetic distance between the four clusters of the South *L. v. vulgaris* Lineage shows that cluster Lvv1 might represent the most distantly related population within the lineage and that clusters Lvv2, Lvv3 and Lvv4 might have differentiated approximately at the same time, being now separated by rivers and lakes (Figure 5).

**TABLE 1:** Pairwise *F*_st_ values for two (A), four (B) and five (C) genetic clusters defined by ADMIXTURE (Alexander et al., 2009).

A	Lvv1	Lva			
Lvv1	‐				
Lva	0.266	‐			

B	Lvv1	Lvv2	Lvv3	Lva	
Lvv1	‐				
Lvv2	0.133	‐			
Lvv3	0.140	0.103	‐		
Lva	0.266	0.311	0.285	‐	

C	Lvv1	Lvv2	Lvv3	Lvv4	Lva
Lvv1	‐				
Lvv2	0.131	‐			
Lvv3	0.157	0.137	‐		
Lvv4	0.154	0.100	0.099	‐	
Lva	0.266	0.310	0.308	0.282	‐

Abbreviations: Lva, *Lissotriton vulgaris ampelensis* lineage; Lvv1–Lvv4, Clusters 1–4 of the South *L. v. vulgaris* Lineage.

### 3.2 Testing the effect of rivers (i.e. the Danube and the Tisza) as barriers to gene flow

**FIGURE 6:**
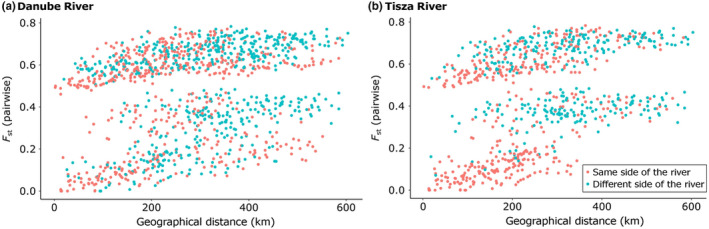
(a) correlation between pairwise *F*_st_ and pairwise geographical distance (km) per population, coloured for being on the same side (pink) or different sides (turquoise) of the river Danube (b) correlation between pairwise *F*_st_ and pairwise geographical distance (km) per population, coloured for being on the same side (pink) or different sides (turquoise) of the river Tisza.

Overall, the pairwise *F*_st_ in relation to the geographical distance of populations on the same side of the river Danube is comparable to the pairwise *F*_st_ in relation to the geographical distance of populations on the opposite sides of the river Danube (Figure 6a). However, it seems that the geographical distance in combination with being on the opposite sides of the river Danube results in relatively‐high pairwise *F*_st_ (there are more turquoise dots on the right top hand of the plot than pink dots; Figure 6a). This effect appears to be stronger for the river Tisza (Figure 6b), as populations on the same side of the river Tisza (pink) have relatively‐low *F*_st_, while there are also populations on the same side of the river Tisza which have high pairwise *F*_st_, comparable to populations on the opposite sides of the river (turquoise) (Figure 6b).

The results of the BEDASSLE analysis show that the river Danube has a very weak effect on population divergence, while the river Tisza has a significant effect on population divergence. According to the model results, being on the opposite sides of the river Tisza is equivalent to being at a ~3000 km distance for *L. vulgaris* populations, while the median geographical distance between populations in our dataset is 279 km (Table 2).

**TABLE 2:** Results of effect sizes for the predictor variables of population position relative to the river Danube and position relative to the river Tisza.

Predictor variable	Mean geographical distance (km; 95% CI)
Position Danube	21.3 (0.01–68.47)
Position Tisza	3016.7 (1385.6–4809.0)

*Note*: The mean geographical distance in km between hypothetical populations necessary to observe a 1‐unit change (km) in the predictor variable.

Abbreviation: 95% CI, 95% confidence intervals.

## 4 DISCUSSION

### 4.1 Distribution and population structure of smooth newt lineages in the Carpathian Basin

Smooth newts are one of the most common urodelans in low‐ and high lands of the forest zone of the temperate belt (Arntzen, Kuzmin, Beebee, et al., 2009; Wielstra et al., 2018). They inhabit a wide variety of freshwater wetland habitats where permanent or temporary standing water is available for breeding (Lõhmus & Linnamägi, 2012; Skorinov et al., 2008). In the present study, we explored the population structure of smooth newts inhabiting the CB and investigated the effect of large rivers (Danube and Tisza) in the diversification of lineages.

Similar to Wielstra et al. (2018), we detected the South *L. v. vulgaris* Lineage distributed in Transdanubia and between the rivers Danube and Tisza, while newly confirming the presence of *L. v. ampelensis* inhabiting the eastern part of the CB. Along the edge of the Carpathian Mountains from Romania to Slovakia, the distribution of *L. v. ampelensis* overlaps with the closely related *L. montandoni*, and their hybridization leads to the replacement of the original mtDNA of *L. montandoni* by introgressed mtDNA of *L. vulgaris* lineages (Babik et al., 2005; Zieliński et al., 2013). Furthermore, extensive gene flow between *L. v. ampelensis* and the North *L. v. vulgaris* Lineage and *L. montandoni* was confirmed (Pabijan et al., 2017; Zieliński et al., 2016). We detected admixture between *L. v. ampelensis* and the South *L. v. vulgaris* Lineage. This observation on the one hand underlines the complex genetic structure of populations in Central Europe, and on the other hand, raises the possibility of gene flow between spatially distinct lineages.

Admixture analysis identified two to five distinct genetic clusters of *L. vulgaris* in the study area. First, if we consider the two clusters scenario, the genetic structure of populations shows a clear separation of the two subspecies (*L. v. vulgaris* [South Lineage] and *L. v. ampelensis*) towards the tributaries of the Danube to the tributaries of the Tisza, with an admixture of lineages through the interfluves of the rivers. Second, according to the fine‐scale scenario, the Transdanubian populations of *L. v. vulgaris* were clustering into four different genetic clusters; in contrast, *L. v. ampelensis* populations remained to be represented by a single cluster with some admixture from the closest cluster of the South *L. v. vulgaris* Lineage (Figure 3c). Thus, the admixed populations of the two subspecies in the interfluves have promoted the existence of a secondary contact zone for Central European smooth newt lineages in the CB. The presence of the four different *L. v. vulgaris* clusters (Lvv1–Lvv4) adds *L. vulgaris* to the list of amphibian species where pronounced genetic diversity is present within the CB. The genetic distance between the four clusters shows that cluster Lvv1 might represent the most distantly related one and that clusters Lvv2–Lvv4 might have differentiated approximately at the same time, being now separated by rivers and lakes (Figures 2 and 5).

Smooth newt populations reached the highest genetic diversity in the Balkans (Pabijan et al., 2015), which served as a refugium for the species during the last ice age (Babik et al., 2005; Pabijan et al., 2015). In the CB, our study found a high level of variation with the greatest genetic diversity observed in the North Hungarian Mountains and the southern edges of the country along the Drava, Danube and Tisza rivers as well as at the confluence of the Sava, Danube and Tisza. While admixture is not pronounced in the North Hungarian Mountains, it cannot be ruled out that high genetic variation at the confluence of the three rivers is due to increased admixture. Similarly, significant structuring of the fire salamander, *Salamandra salamandra* populations has been described from the North Hungarian Mountains, which are situated at the edge of the Carpathians and which might have provided glacial refugia for the fire salamander populations (Vörös et al., 2017). The confluence of the Sava, Danube and Tisza rivers contributed to the highest diversity for the Danube crested newt, *Triturus dobrogicus*, populations also supporting the presence of glacial refugia (Vörös et al., 2016). The coincidences with the genetic pattern of *L. vulgaris* found in this study (i.e. high genetic diversity in the North Hungarian Mountains as well as at the confluence of the Sava, Danube and Tisza rivers) suggest that one or multiple northerly refugia in the CB cannot be ruled out for the smooth newt as well.

### 4.2 Effects of landscape elements (i.e. rivers) on the population structure of *L. vulgaris*

It was a long‐standing belief that smooth newts have a limited dispersal ability between 50 and 182 m (Bell, 1977; Dolmen, 1981); however, recent studies suggested greater dispersal distance of up to 2 km for individuals (Schmidt et al., 2006; Tóth et al., 2021). Moreover, females usually return to their natal pond to reproduce (Bell, 1977). Therefore, the limited dispersal abilities on land, the high fidelity towards breeding habitats and, most importantly, the avoidance of habitats associated with flowing water (Lõhmus & Linnamägi, 2012) could restrict the dispersion of smooth newt lineages.

Our findings on population structure suggest an underlying barrier effect of one of the large rivers behind the observed pattern of population structure of smooth newt lineages in the CB. The river Tisza stands as a barrier to *L. v. ampelensis* to disperse towards the West and to the South *L. v. vulgaris* Lineage towards the East. The four South *L. v. vulgaris* Lineage clusters distributed in the southern Transdanubia (Lvv1), the northern Transdanubia (Lvv2) and the Danube–Tisza Interfluve (Lvv3 and Lvv4) show that landscape features—such as the river Danube or the Lake Balaton—might shape also the population structure within *L. v. vulgaris* in the CB. The interfluve of the rivers (i.e. from the Great Plains of Hungary towards northern Serbia) serves as a contact zone for lineages, which was confirmed by the presence of admixed populations in the area. This population structure is corresponding to the distributional pattern of the Danube crested newts that showed a weak but significant genetic structure between tributaries of the three main river systems of Danube, Sava, and Tisza in the CB (Vörös & Arntzen, 2010). Nevertheless, the Danube crested newts are also present in rivers and their associated oxbows (Arntzen, Kuzmin, Jehle, et al., 2009); therefore rivers, especially under flooding conditions may not be as strict barriers for dispersal, and thereby for genetic structuring as presumably did in smooth newts.

## 5 CONCLUDING REMARKS

European newts from *L. vulgaris* sensu lato were proposed to form a speciation continuum, influenced by the most extensive genetic exchange among lineages inhabiting Central Europe. Gene flow between the two morphologically cryptic North *L. v. vulgaris* and South *L. v. vulgaris* Lineages and the neighbouring *L. v. ampelensis* were observed East and South of the CB, but population structure and lineage diversification within this biogeographically key region were not explored before. With the help of genomic data, we extended our knowledge on gene flow between the South *L. v. vulgaris* Lineage and *L. v. ampelensis*, confirming a ca. 400 km long contact zone along the river Tisza and in the interfluves of the Danube and Tisza rivers in Central Europe. Moreover, we identified the river Tisza as an important geographical barrier limiting gene flow between the lineages. The *L. v. vulgaris* showed a complex population genetic pattern in the CB that led us to hypothesize one or more Pleistocene refugia for the species.

## AUTHOR CONTRIBUTIONS

**Dávid Herczeg:** Conceptualization (supporting); writing – original draft (lead); writing – review and editing (lead). **Gemma Palomar:** Formal analysis (lead); methodology (equal); software (lead); writing – review and editing (equal). **Piotr Zieliński:** Formal analysis (supporting); methodology (equal); writing – review and editing (equal). **Isolde van Riemsdijk:** Formal analysis (supporting); methodology (equal); writing – review and editing (equal). **Wiesław Babik:** Methodology (equal); writing – review and editing (equal). **Róbert Dankovics:** Investigation (equal). **Bálint Halpern:** Investigation (equal). **Milena Cvijanović:** Investigation (equal). **Judit Vörös:** Conceptualization (lead); funding acquisition (lead); supervision (lead); writing – original draft (supporting); writing – review and editing (equal).

## CONFLICT OF INTEREST STATEMENT

The authors declare no conflicts of interest.

## Supporting information


Data S1

## Data Availability

The .vcf file with filtered SNP data was deposited to Figshare Digital Repository doi: https://doi.org/10.6084/m9.figshare.23922108.v1.
