# Supplementary material for: Genomic analysis reveals complex population structure within the smooth newt, Lissotriton vulgaris, in Central Europe
Source: Ecol Evol. 2023 Aug 31;13(9):e10478. doi: 10.1002/ece3.10478 (PMC10469019; doi:10.1002/ece3.10478)

Electronic Supplementary Material

**Genomic analysis reveals complex population structure within the smooth newt, *Lissotriton vulgaris* in Central Europe**

Dávid Herczeg^1,2,*^, Gemma Palomar^3,4^, Piotr Zieliński^4^, Isolde van Riemsdijk^5^, Wiesław Babik^4^, Róbert Dankovics^6^, Bálint Halpern^1,2,7^, Milena Cvijanović^8^_,_ Judit Vörös^9^

^1^ELKH-ELTE-MTM Integrative Ecology Research Group, Budapest, Hungary

^2^Department of Systematic Zoology and Ecology, Institute of Biology, ELTE Eötvös Loránd University, Budapest, Hungary

^3^Department of Genetics, Physiology, and Microbiology, Complutense University of Madrid, Madrid, Spain

^4^Institute of Environmental Sciences, Faculty of Biology, Jagiellonian University, Kraków, Poland

^5^Plant Evolutionary Ecology Group, University of Tübingen, Tübingen, Germany

^6^Savaria Museum, Szombathely, Hungary

^7^MME Birdlife Hungary, Budapest, Hungary

^8^Institute for Biological Research „Siniša Stanković”, National Institute of the Republic of Serbia, University of Belgrade, Belgrade, Serbia

^9^Department of Zoology, Hungarian Natural History Museum, Budapest, Hungary

** corresponding author*

Email: herczegdavid88@gmail.com

Table S1. Population ID (Pop. ID), number of individuals (N), country, locality, WGS84 geocoordinates of 58 *L. vulgaris* populations. Lat = latitude; Long = longitude

| \| **Pop. ID** \| **N** \| **Country** \| **Locality** \| **Lat** \| **Long** \| \| --- \| --- \| --- \| --- \| --- \| --- \| \| 1 \| 9 \| Hungary \| Kardoskút. Fehér Lake \| 46.472 \| 20.616 \| \| 2 \| 1 \| Hungary \| Szabadkígyós \| 46.577 \| 21.141 \| \| 3 \| 7 \| Hungary \| Parádfürdő. Pisztrángos Lake \| 47.882 \| 20.013 \| \| 4 \| 15 \| Hungary \| Babatpuszta \| 47.624 \| 19.382 \| \| 5 \| 12 \| Hungary \| Budapest. Mocsáros dűlő \| 47.576 \| 19.042 \| \| 6 \| 1 \| Hungary \| Budapest. Pesterzsébet \| 47.429 \| 19.134 \| \| 7 \| 1 \| Hungary \| Ócsa \| 47.262 \| 19.232 \| \| 8 \| 10 \| Hungary \| Kunpeszér \| 47.023 \| 19.288 \| \| 9 \| 1 \| Hungary \| Váralja. Farkas Valley \| 46.246 \| 18.419 \| \| 10 \| 5 \| Hungary \| Gyékényes. Lankóci Forest \| 46.233 \| 17.051 \| \| 11 \| 5 \| Hungary \| Őriszentpéter \| 46.877 \| 16.436 \| \| 12 \| 2 \| Hungary \| Szakonyfalu \| 46.908 \| 16.238 \| \| 13 \| 1 \| Hungary \| Sárvár. Rába oxbow \| 47.275 \| 16.963 \| \| 14 \| 15 \| Hungary \| Bakonybél \| 47.268 \| 17.694 \| \| 15 \| 11 \| Hungary \| Magyargencs \| 47.369 \| 17.296 \| \| 16 \| 1 \| Hungary \| Pusztacsalád-Cirák \| 47.484 \| 16.947 \| \| 17 \| 1 \| Hungary \| Sopron. Lővérek \| 47.653 \| 16.531 \| \| 18 \| 1 \| Slovakia \| Bratislava-Dúbravka \| 48.197 \| 17.038 \| \| 19 \| 1 \| Slovakia \| NPR Šúr \| 48.228 \| 17.205 \| \| 20 \| 1 \| Slovakia \| Parížské močiare \| 47.858 \| 18.510 \| \| 21 \| 1 \| Slovakia \| Lipníky \| 49.065 \| 21.424 \| \| 22 \| 1 \| Ukraine \| Dertsen \| 48.355 \| 22.650 \| \| 23 \| 1 \| Ukraine \| Lypcha \| 48.279 \| 23.380 \| \| 24 \| 1 \| Hungary \| Erdőhorváti \| 48.275 \| 21.347 \| \| 25 \| 1 \| Hungary \| Olaszliszka \| 48.230 \| 21.414 \| \| 26 \| 1 \| Hungary \| Harsány \| 48.013 \| 20.763 \| \| 27 \| 1 \| Hungary \| Tiszacsege \| 47.707 \| 20.949 \| \| 28 \| 1 \| Hungary \| Bátaszék \| 46.200 \| 18.823 \| \| 29 \| 1 \| Hungary \| Zalaháshágy1 \| 46.909 \| 16.611 \| \| 30 \| 1 \| Hungary \| Zalaháshágy2 \| 46.926 \| 16.622 \| \| 31 \| 1 \| Slovenia \| Radenci \| 46.644 \| 16.040 \| \| 32 \| 1 \| Croatia \| Peteranec \| 46.194 \| 16.887 \| \| 33 \| 1 \| Serbia \| Makova Sedmica \| 46.148 \| 19.674 \| \| 34 \| 1 \| Serbia \| Selevenjske Pustare \| 46.147 \| 19.906 \| \| 35 \| 1 \| Serbia \| Banatsko Veliko Selo \| 45.827 \| 20.594 \| \| 36 \| 1 \| Serbia \| Glušci \| 44.994 \| 19.729 \| \| 37 \| 1 \| Serbia \| Orid \| 44.712 \| 19.797 \| \| 38 \| 1 \| Serbia \| Opovo \| 45.061 \| 20.436 \| \| 39 \| 1 \| Serbia \| Kovacica \| 45.141 \| 20.561 \| \| 40 \| 1 \| Serbia \| Umka \| 44.681 \| 20.320 \| \| 41 \| 1 \| Serbia \| Djurinci1 \| 44.512 \| 20.632 \| \| 42 \| 1 \| Serbia \| Djurinci2 \| 44.494 \| 20.653 \| \| 43 \| 1 \| Serbia \| Sisevac \| 43.957 \| 21.584 \| \| 44 \| 1 \| Serbia \| Zlot \| 44.011 \| 21.967 \| \| 45 \| 1 \| Serbia \| Majdanpek \| 44.434 \| 21.974 \| \| 46 \| 1 \| Serbia \| Duboka \| 44.569 \| 21.766 \| \| 47 \| 1 \| Serbia \| Vatin \| 45.230 \| 21.244 \| \| 48 \| 1 \| Romania \| Mercina \| 45.063 \| 21.536 \| \| 49 \| 1 \| Romania \| Jiu Gorge \| 45.252 \| 23.418 \| \| 50 \| 1 \| Romania \| Zeicani \| 45.506 \| 22.709 \| \| 51 \| 1 \| Romania \| Cărpiniş \| 45.878 \| 23.026 \| \| 52 \| 1 \| Romania \| Pecica1 \| 46.134 \| 21.052 \| \| 53 \| 1 \| Romania \| Arieseni \| 46.465 \| 22.783 \| \| 54 \| 1 \| Romania \| Valea Ierii \| 46.650 \| 23.355 \| \| 55 \| 1 \| Romania \| Bogdana \| 47.028 \| 23.031 \| \| 56 \| 1 \| Romania \| Peştiş1 \| 47.089 \| 22.390 \| \| 57 \| 1 \| Romania \| Mănăşturel \| 47.189 \| 23.927 \| \| 58 \| 1 \| Romania \| Pomi \| 47.694 \| 23.320 \| |  |  |  |  |  |
| --- | --- | --- | --- | --- | --- | --- | --- | --- | --- | --- | --- | --- | --- | --- | --- | --- | --- | --- | --- | --- | --- | --- | --- | --- | --- | --- | --- | --- | --- | --- | --- | --- | --- | --- | --- | --- | --- | --- | --- | --- | --- | --- | --- | --- | --- | --- | --- | --- | --- | --- | --- | --- | --- | --- | --- | --- | --- | --- | --- | --- | --- | --- | --- | --- | --- | --- | --- | --- | --- | --- | --- | --- | --- | --- | --- | --- | --- | --- | --- | --- | --- | --- | --- | --- | --- | --- | --- | --- | --- | --- | --- | --- | --- | --- | --- | --- | --- | --- | --- | --- | --- | --- | --- | --- | --- | --- | --- | --- | --- | --- | --- | --- | --- | --- | --- | --- | --- | --- | --- | --- | --- | --- | --- | --- | --- | --- | --- | --- | --- | --- | --- | --- | --- | --- | --- | --- | --- | --- | --- | --- | --- | --- | --- | --- | --- | --- | --- | --- | --- | --- | --- | --- | --- | --- | --- | --- | --- | --- | --- | --- | --- | --- | --- | --- | --- | --- | --- | --- | --- | --- | --- | --- | --- | --- | --- | --- | --- | --- | --- | --- | --- | --- | --- | --- | --- | --- | --- | --- | --- | --- | --- | --- | --- | --- | --- | --- | --- | --- | --- | --- | --- | --- | --- | --- | --- | --- | --- | --- | --- | --- | --- | --- | --- | --- | --- | --- | --- | --- | --- | --- | --- | --- | --- | --- | --- | --- | --- | --- | --- | --- | --- | --- | --- | --- | --- | --- | --- | --- | --- | --- | --- | --- | --- | --- | --- | --- | --- | --- | --- | --- | --- | --- | --- | --- | --- | --- | --- | --- | --- | --- | --- | --- | --- | --- | --- | --- | --- | --- | --- | --- | --- | --- | --- | --- | --- | --- | --- | --- | --- | --- | --- | --- | --- | --- | --- | --- | --- | --- | --- | --- | --- | --- | --- | --- | --- | --- | --- | --- | --- | --- | --- | --- | --- | --- | --- | --- | --- | --- | --- | --- | --- | --- | --- | --- | --- | --- | --- | --- | --- | --- | --- | --- | --- | --- | --- | --- | --- | --- | --- | --- | --- | --- | --- | --- | --- | --- | --- | --- | --- | --- | --- | --- | --- | --- | --- | --- | --- | --- | --- | --- | --- | --- | --- | --- | --- | --- | --- | --- | --- |

Fig. S1. Expected heterozygosity (HE) across the 58 *L. vulgaris* populations computed in Arlequin 3.5.


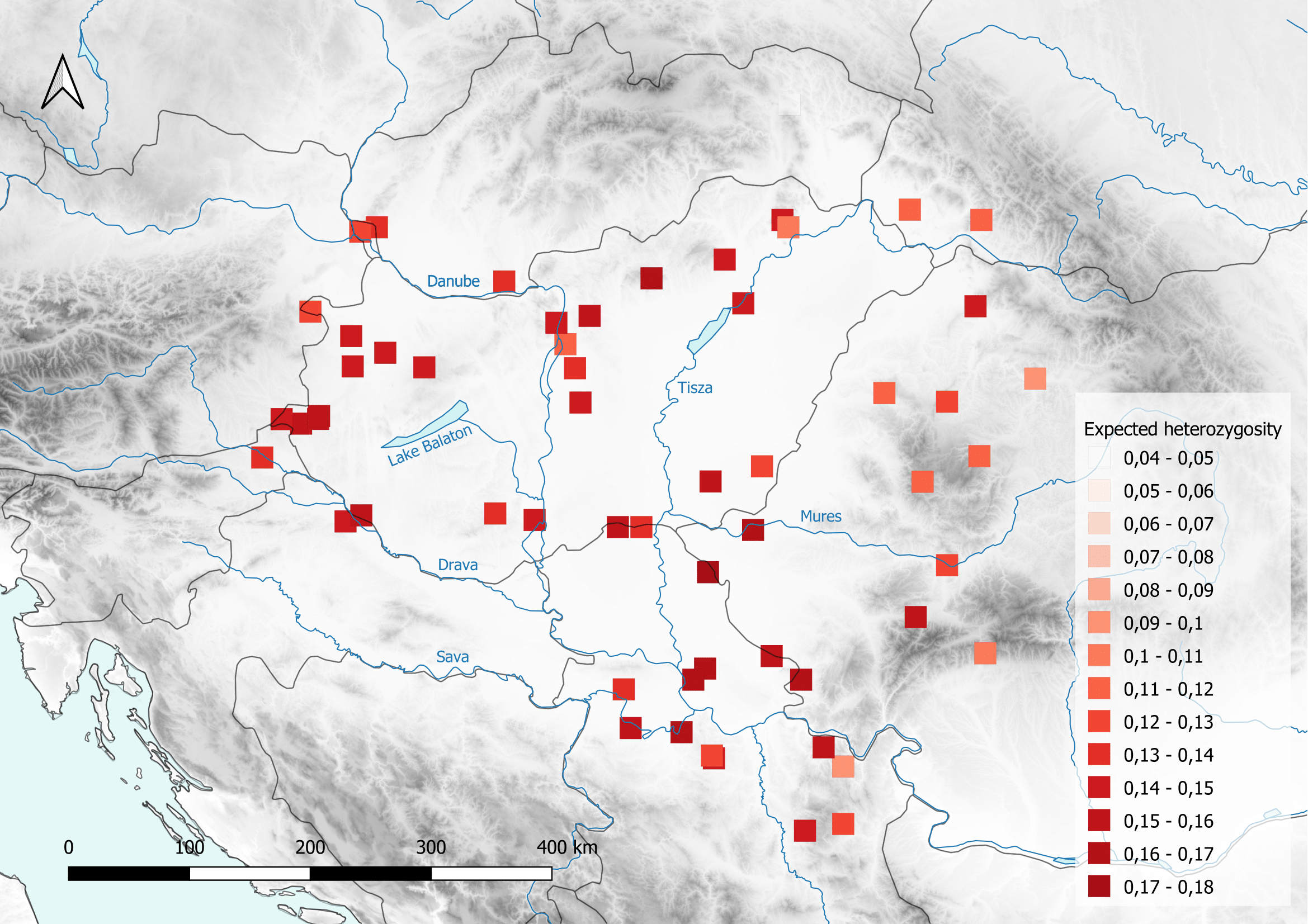


Fig. S2. **A:** Trace plots showing appropriate mixing of the model for the variables including the 200 sample burn-in. After 200 samples, the predictor variables all demonstrated appropriate model mixing. **B:** We ran 1000 posterior predictive samples to determine that the model was a good fit for the data. Red dots are observed values and black dots are the distribution of predicted values from the model, indicating that the model accurately predicts the data distribution.


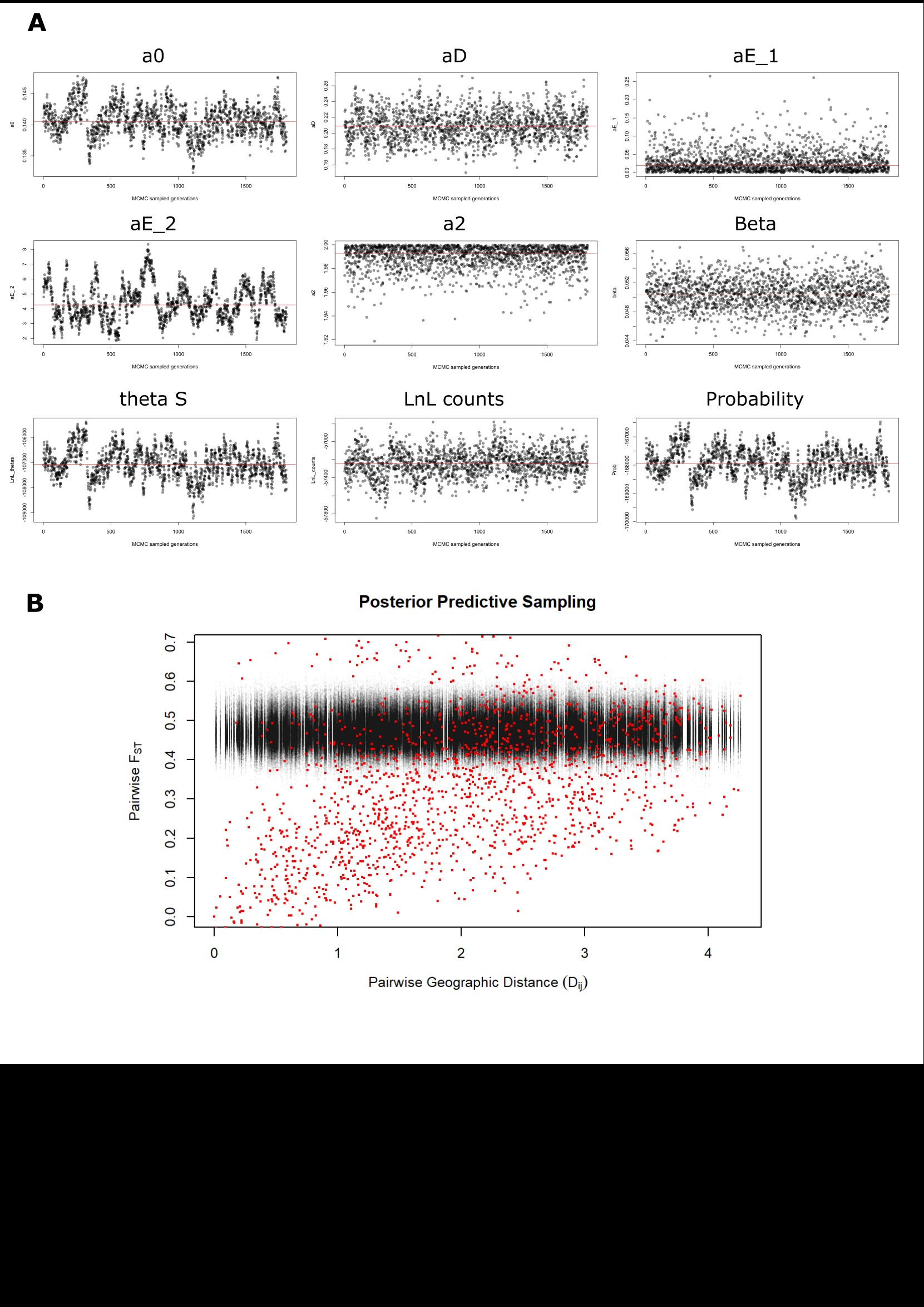

Supplement: Supplementary file 1 — Data S1 [file ECE3-13-e10478-s001.docx]
